# Development and validation of a nomogram for predicting recurrence in epithelial ovarian cancer after primary cytoreductive surgery: A single-center retrospective study

**DOI:** 10.12669/pjms.42.5.14519

**Published:** 2026-05

**Authors:** Weifeng Zhang, Yan Shu, Zeqiu Wan, Shuying Yang

**Affiliations:** 1 Weifeng Zhang Department of Gynecology, Huzhou Maternity & Child Health Care Hospital,Huzhou, Zhejiang Province 313000, P.R. China; 2 Yan Shu Department of Ultrasound, Huzhou Maternity & Child Health Care Hospital,Huzhou, Zhejiang Province 313000, P.R. China; 3 Zeqiu Wan Department of Gynecology, Huzhou Maternity & Child Health Care Hospital,Huzhou, Zhejiang Province 313000, P.R. China; 4 Shuying Yang, Huzhou Maternity & Child Health Care Hospital,Huzhou, Zhejiang Province 313000, P.R. China

**Keywords:** Cytoreductive surgery, Epithelial ovarian cancer, Primary, Prediction, Recurrence

## Abstract

**Objective:**

To explore the risk factors for recurrence of epithelial ovarian cancer (EOC) after primary cytoreductive surgery, and to develop and validate a risk prediction model based on the identified factors.

**Methodology:**

Clinical data of 213 patients with epithelial ovarian cancer (EOC) who underwent primary cytoreductive surgery (CRS) in Huzhou Maternity & Child Health Care Hospital, China from January 2020 to July 2025 were retrospectively collected. Patients were randomly divided into a training (n=128) and a validation cohort (n=85) at a ratio of 6:4. The Least Absolute Shrinkage and Selection Operator (LASSO) regression and multivariable logistic regression were utilized for feature selection in the training cohort to construct a nomogram for predicting postoperative recurrence. The discriminative performance and calibration of the nomogram were evaluated in both cohorts utilizing the area under the receiver operating characteristic curve (AUC) and the Hosmer-Lemeshow test.

**Results:**

The EOC recurrence rate was 40.8% (87/213). The analysis identified six independent risk factors for predicting recurrence, including International Federation of Gynecology and Obstetrics (FIGO) stage, differentiation grade, residual lesions, positive ascites cytology, carbohydrate antigen 125 (CA125) positive, and positive Risk of Ovarian Malignancy Algorithm (ROMA) index. The nomogram model demonstrated sufficient predictive accuracy, with AUC values of 0.861 (95%CI: 0.795-0.928) and 0.801 (95%CI: 0.697-0.904) in the training and validation cohorts, respectively. The results of the H-L test showed good fitness.

**Conclusions:**

The nomogram model developed in this study exhibits good predictive performance and applicability, and can be used to screen for the risk of recurrence in this population.

## INTRODUCTION

Epithelial ovarian cancer (EOC) remains a leading cause of death from gynecologic cancers in women worldwide.^1^ Despite advances in early detection and treatment (including surgery and chemotherapy), the overall prognosis of EOC patients remains poor, primarily due to high recurrence rates and the lack of effective long-term treatment strategies.^2^ Most EOC cases are diagnosed at an advanced stage, with over 70% of patients presenting with extensive metastases at the time of diagnosis.^3^ For patients with advanced-stage (Stage III and IV) ovarian cancer, the five-year survival rate is particularly low, which highlights the need for more accurate prognostic tools to guide treatment and improve survival outcomes.^4^

Primary cytoreductive surgery (CRS) is the cornerstone of treatment for advanced ovarian cancer, aiming to reduce tumor burden and enhance the efficacy of subsequent chemotherapy.^5^ However, recurrence remains a significant challenge, with up to 80% of patients experiencing recurrence within five years.^6^ Understanding the factors influencing recurrence risk and developing reliable prediction models may improve treatment strategies by enabling personalized therapy, thereby optimizing survival outcomes.^7^

The prognostic factors for predicting recurrence after CRS include tumor grade, histological subtype, and the presence of residual lesions after surgery.^8^,^9^ Molecular markers, such as the carbohydrate antigen 125 (CA125) and genetic mutations in key oncogenes, such as TP53 and BRCA1/2, were also shown to be reliable predictors of the recurrence risk.^10^,^11^ Additionally, growing research on the tumor microenvironment, immune response, and methylated driver gene expression has provided further insights into the mechanisms underlying tumor recurrence.^12^

Over the past decade, nomogram models have shown promising results in various cancer types.^13^ However, while several studies have attempted to use clinicopathological data and develop predictive nomograms for EOC, the specificity and accuracy of these models remain inconsistent across different patient cohorts.^14^

This study aimed to develop and validate a model for predicting recurrence in EOC patients after primary CRS. Such a model can potentially serve as a valuable clinical tool for stratifying patients based on recurrence risk, guiding treatment decisions, and enhancing the overall quality of patient care.

## METHODOLOGY

The study retrospectively collected clinical data of patients with EOC who underwent primary CRS between January 2020 to July 2025.

### Ethical approval:

This study was approved by the Institutional Review Board of Huzhou Maternity & Child Health Care Hospital (Approval No. 2021-R-002; November 16, 2021).

### Inclusion criteria:

- Diagnosis of primary epithelial ovarian cancer (encompassing major histological subtypes including serous, endometrioid, clear cell, and mucinous carcinomas) confirmed by imaging modalities (such as color Doppler ultrasound and computed tomography) alongside histopathological and cytological examinations.
- No prior radiotherapy, targeted therapy, or previous ovarian cancer-directed surgery before cytoreductive surgery; neoadjuvant chemotherapy, if administered, was recorded and analyzed as a baseline treatment-related variable.
- All enrolled patients underwent primary CRS followed by guideline-concordant platinum-based systemic chemotherapy, thereby minimizing treatment-related heterogeneity within the cohort.
- Karnofsky Performance Status (KPS) score ≥ 60.
- Availability of complete clinical data with a minimum postoperative follow-up of three years.

### Exclusion criteria:

- Severe hepatic or renal dysfunction.
- Acute hematological system infections.
- A history of other malignancies within the previous five years.
- Concomitant severe autoimmune diseases.

### Criteria for definite diagnosis of EOC Recurrence:

Tumor recurrence within three years after surgery was recorded. Recurrence was evaluated based on imaging examinations and serum tumor marker tests. Recurrence was confirmed when imaging showed enlargement of residual lesions or the appearance of new lesions, together with clinical evidence and/or pathological confirmation by image-guided biopsy or surgical biopsy when available.

### Collected indicators:

Utilizing the date of primary CRS as the index time point, preoperative baseline, perioperative, and postoperative follow-up data were systematically extracted within the predefined time frames. All information was sourced from the hospital’s electronic medical records (including clinical and surgical records), pathological reports, laboratory information systems (for tumor markers), and imaging data. Data were independently selected and cross-checked by two researchers. Any inconsistencies were assessed by a third party. The study population, inclusion/exclusion criteria, follow-up period, and recurrence determination method were as described in this study protocol and main text (follow-up for at least three years or until recurrence events occur, whichever comes first; recurrence determined based on imaging progression and combined with clinical/pathological evidence).

### Primary outcome:

The primary outcome was “postoperative recurrence”. Recurrence was defined as imaging evidence of enlargement of the primary lesion or emergence of new lesions, confirmed by clinical and/or pathological/cytological findings. Patients without recurrence were censored at the end of the follow-up period. This outcome served as the dependent variable in the prediction model, which was used for model development in the training cohort and for model evaluation in the validation cohort.

### Candidate Predictive Variables:

### Demographic and general data:

age (on the day of surgery), body mass index (BMI); educational level was recorded based on past medical history.

### Oncological Characteristics:

- FIGO stage (I–IV, determined by postoperative pathology/comprehensive assessment).
- Histological type (serous or non-serous).
- Histopathological grade/differentiation degree.
- Ascites cytology (negative/positive).

### Surgical Information:

- Surgical approach (laparotomy, laparoscopy, or converted to laparotomy).
- Postoperative residual lesions (no macroscopic residue or maximum diameter ≤ 1 cm, and > 1 cm; the maximum residual diameter was recorded for description).
- Lymph node dissection (performance and scope were based on surgical records).
- Systemic Treatment Information.
- Neoadjuvant chemotherapy (whether received, number of cycles, and interval from chemotherapy to surgery).
- Postoperative first-line chemotherapy regimen (intravenous chemotherapy or intravenous + intraperitoneal chemotherapy); the start time of the first chemotherapy cycle was recorded to verify procedural consistency.

### Serum Tumor Markers:

- CA125, HE4, and ROMA index. Original test results and laboratory interpretations (negative/positive, based on reports from the hospital’s clinical laboratory) were extracted.

All the above variables were clinically accessible in previous practice and served as candidate indicators for univariate comparison, feature selection, and multivariate modeling in this study. After selection via LASSO and multivariate logistic regression, the independent risk factors included in the final nomogram were: FIGO stage, differentiation degree, residual lesions, ascites cytology, positive CA125, and positive ROMA index.

### Statistical analysis:

The study dataset was randomly divided into a training cohort and a validation cohort at a 6:4 ratio using IBM SPSS Statistics (Version 26.0, IBM Corp, Armonk, NY, USA). The training cohort was used for developing the nomogram, while the validation cohort was utilized for internal validation.

Visual methods (histograms and Q-Q plots) and analytical methods (Kolmogorov-Smirnov and Shapiro-Wilk tests) were used to assess the normality of continuous variables. Non-normally distributed continuous variables were presented as medians and interquartile ranges, with intergroup comparisons performed using the Mann-Whitney U test. Categorical variables were expressed as counts and percentages, and compared using the chi-square test.

To identify independent prognostic factors and mitigate the risk of overfitting, the Least Absolute Shrinkage and Selection Operator (LASSO) regression was employed. The optimal penalty parameter (λ) was determined using 10-fold cross-validation based on the minimum binomial deviance. The minimum criteria (λ_min_) was applied to select the most predictive features. The variables identified through LASSO were subsequently incorporated into a multivariable logistic regression model to construct the nomogram. To ensure the statistical robustness of this multivariable model, the events-per-variable ratio was rigorously evaluated. Given 87 recorded recurrence events and a total of eight predictor parameters—comprising three dummy variables derived from the multi-level categorical variable (International Federation of Gynecology and Obstetrics stage) alongside five binary variables—the calculated events-per-variable ratio was approximately 10.9. This value satisfies the traditional methodological recommendation of maintaining at least 10 events per parameter, thereby minimizing the potential for overfitting.

The predictive efficacy of the developed model was evaluated in both cohorts using the receiver operating characteristic curve and the calibration curve. The area under the curve ranges from 0.50 to 1.00, with higher values indicating superior discriminative performance. Calibration plots were generated to assess the agreement between the predicted probabilities and the actual observed recurrence outcomes. Finally, decision curve analysis was conducted to quantify the clinical net benefit and utility of the nomogram across a range of threshold probabilities. A two-sided p-value < 0.05 was considered statistically significant.

## RESULTS

### Baseline characteristics and univariate analysis:

A total of 213 patients were included in this study, with 128 in the training cohort and 85 in the validation cohort. The recurrence rates observed in the training cohort and validation cohort were 39.8% (51/128) and 42.4% (36/85), respectively. There was no statistically significant difference in the recurrence rate between the two cohorts (*χ²=0.133, P=0.715*). The baseline characteristics of the training cohort and validation cohort are described in Table-I.

**Table-I:** Baseline characteristics of patients.

Characteristics	Subcategory	Training cohort (n=128)	Validation cohort (n=85)	t/χ²	P
Age (years)		56.2 ± 6.9	55.4 ± 8.2	0.808	0.420
BMI (kg/m²)		21.8 ± 2.0	22.2 ± 2.4	-1.529	0.128
Education Level				0.241	0.623
	Junior high and below	85 (66.4%)	60 (70.6%)		
	High school and above	43 (33.6%)	25 (29.4%)		
FIGO Stage				2.451	0.484
	Stage I	14 (10.9%)	15 (17.6%)		
	Stage II	29 (22.7%)	17 (20.0%)		
	Stage III	63 (49.2%)	42 (49.4%)		
	Stage IV	22 (17.2%)	11 (12.9%)		
Pathological Grade				2.024	0.155
	Grade I-II	43 (33.6%)	20 (23.5%)		
	Grade III	85 (66.4%)	65 (76.5%)		
Histology				0.083	0.773
	Serous cancer	88 (68.8%)	56 (65.9%)		
	Non-serous cancer	40 (31.2%)	29 (34.1%)		
Differentiation				<0.001	>0.999
	Poorly differentiated	35 (27.3%)	23 (27.1%)		
	Moderately/Highly differentiated	93 (72.7%)	62 (72.9%)		
Residual Lesion				<0.001	0.992
	≤1 cm	86 (67.2%)	58 (68.2%)		
	>1 cm	42 (32.8%)	27 (31.8%)		
Ascites Cytology				0.854	0.355
	Negative	96 (75.0%)	58 (68.2%)		
	Positive	32 (25.0%)	27 (31.8%)		
Chemotherapy Regimen				1.659	0.198
	Intravenous	89 (69.5%)	51 (60.0%)		
	Intravenous + Intraperitoneal	39 (30.5%)	34 (40.0%)		
Neoadjuvant Chemotherapy				0.496	0.481
	No	100 (78.1%)	62 (72.9%)		
	Yes	28 (21.9%)	23 (27.1%)		
Surgical Approach				1.349	0.246
	Laparotomy	44 (34.4%)	22 (25.9%)		
	Laparoscopy	84 (65.6%)	63 (74.1%)		
Lymph Node Dissection				1.516	0.218
	No	51 (39.8%)	26 (30.6%)		
	Yes	77 (60.2%)	59 (69.4%)		
CA125 Positive				0.709	0.400
	Negative	75 (58.6%)	44 (51.8%)		
	Positive	53 (41.4%)	41 (48.2%)		
HE4 Positive				0.014	0.906
	Negative	73 (57.0%)	50 (58.8%)		
	Positive	55 (43.0%)	35 (41.2%)		
ROMA Index Positive				0.381	0.537
	Negative	64 (50.0%)	38 (44.7%)		
	Positive	64 (50.0%)	47 (55.3%)		
Recurrence				0.133	0.715
	No	77 (60.2%)	49 (57.6%)		
	Yes	51 (39.8%)	36 (42.4%)		

### Predictors of Postoperative Recurrence:

**Fig.1:**
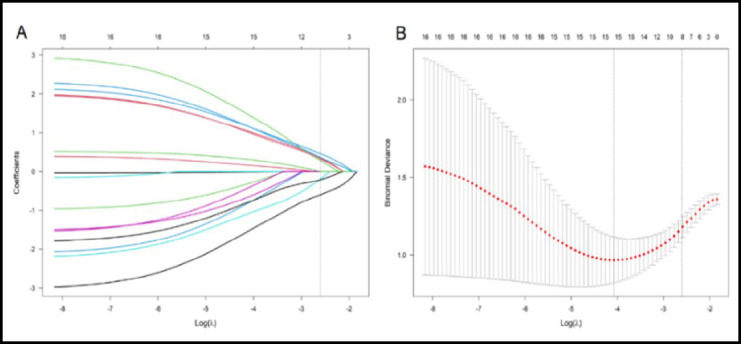
LASSO Coefficient Curves for Postoperative Recurrence. A. Each curve represents the coefficient trajectory of individual variables. The vertical axis denotes the coefficient value, the lower horizontal axis represents log(λ), and the upper horizontal axis indicates the number of non-zero coefficients at the corresponding log(λ). B. The optimal penalty parameter (λ) was selected following a 10-fold cross-validation based on binomial deviance. The left vertical dashed line delineates the optimal value determined by the minimum criteria (λ_min_), corresponding to the selected prognostic factors.

Predictors of postoperative recurrence were first selected via LASSO regression. Variables were centralized and normalized through 10-fold cross-validation (Fig.1). The selected predictors included FIGO stage, differentiation degree, residual lesions, ascites cytology, lymph node dissection, positive CA125, HE4 positive, and ROMA index positive. These eight predictors were then included as independent risk variables, and a predictive model was constructed using multivariate logistic regression (Table-II). The results showed that FIGO stage, differentiation degree, residual lesions, positive ascites cytology, CA125 positive, and ROMA index positive were independent risk factors for postoperative recurrence.

**Table-II:** Multivariate logistic regression analysis of predictors selected via LASSO regression in the development cohort.

Independent variables	B	OR (95% CI)	P
FIGO Stage I	0.852	2.345 (0.298~18.471)	0.418
FIGO Stage II	1.938	6.942 (1.077~44.731)	0.042
FIGO Stage III	3.187	24.226 (2.895~202.707)	0.003
Moderately/Highly differentiated	-1.191	0.304 (0.101~0.914)	0.034
Residual lesions >1 cm	1.314	3.721 (1.329~10.416)	0.012
Positive ascites cytology	1.237	3.445 (1.068~11.117)	0.038
CA125 positive	1.653	5.224 (1.838~14.847)	0.002
ROMA index positive	1.468	4.340 (1.579~11.933)	0.004

### Nomogram for postoperative recurrence:

**Fig.2:**
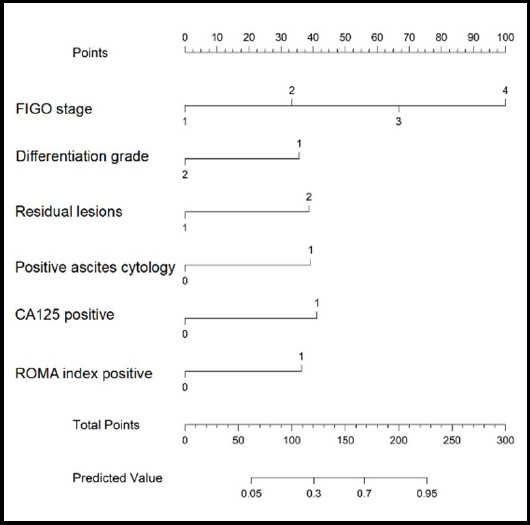
Nomogram of postoperative recurrence. Each level of the predictor variable represents a specific score. The total score is generated by summarizing the scores of each predictor variable. The total score corresponds to the probability of recurrence.

A nomogram model for predicting postoperative recurrence risk was constructed based on the six independent risk factors mentioned above (Fig.2). A total score was calculated by summing the points assigned to each respective predictor within the nomogram. This cumulative score corresponds to the predicted probability of postoperative recurrence for individual patients.

### Calibration and Validation of the Nomogram:

**Fig.3:**
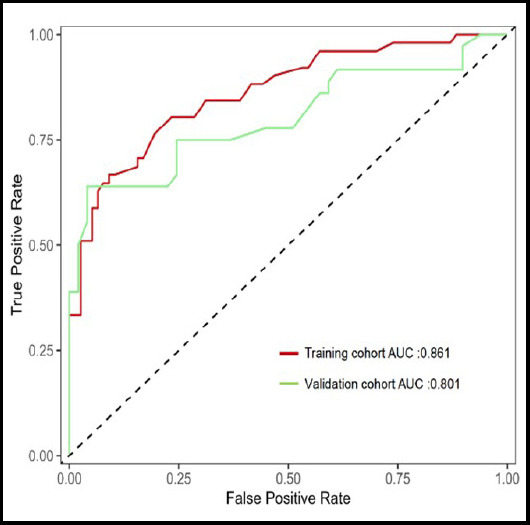
ROC curve and AUC of the prediction model. ROC: receiver operating characteristic, AUC: area under the curve.

**Fig.4:**
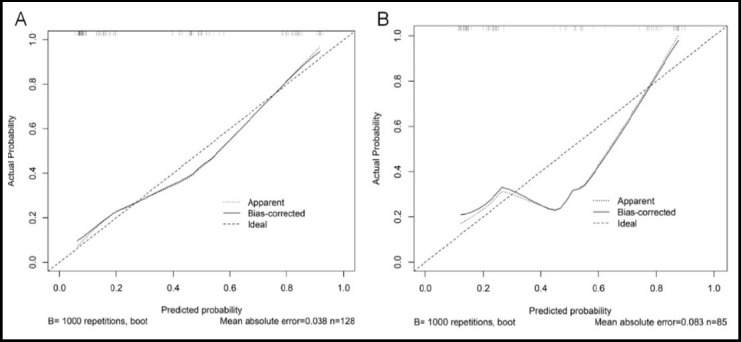
Calibration plots of the prediction model. A. Calibration plot for the training cohort. B. Calibration plot for the validation cohort. The x-axis represents the predicted probability of recurrence, and the y-axis represents the actual observed recurrence. The diagonal dashed line signifies a perfect prediction by an ideal model. The solid line illustrates the performance of the developed nomogram; a closer alignment between the solid line and the diagonal dashed line indicates superior predictive accuracy.

**Fig.5:**
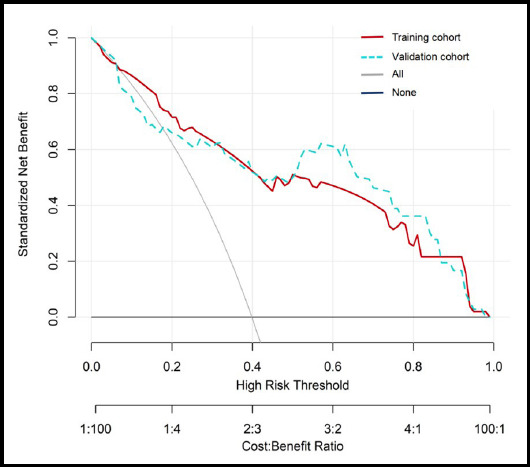
DCA of Nomogram. DCA: decision curve analysis.

As demonstrated by the Hosmer-Lemeshow test, the training cohort showed *a χ2 value of 4.855 (P = 0.773), and the validation cohort showed a χ2 value of 2.621 (P = 0.956*), indicating that the predicted outcomes were close to the observed outcomes. The ROC curve in the training cohort demonstrated good discriminative ability (AUC: 0.861; 95% CI: 0.795-0.928). The discriminative performance of the model was validated in the validation cohort (AUC: 0.801; 95% CI: 0.697 - 0.904) (Fig.3). Calibration curve analysis demonstrated good consistency between the predicted probability and the observed postoperative recurrence in both the training and validation cohorts (Fig.4). Decision curve analysis (DCA) demonstrated the clinical utility of the model (Fig.5).

## DISCUSSION

The primary novelty of this study lies in the development of an integrated prognostic nomogram specifically targeting early recurrence (within three years) following primary cytoreductive surgery for epithelial ovarian cancer. A major strength of this predictive model is its reliance on readily accessible perioperative clinical and laboratory data, ensuring high practical applicability in routine clinical settings. By systematically integrating traditional anatomical staging and histopathological characteristics with the Risk of Ovarian Malignancy Algorithm index and a critical therapeutic factor (residual disease), this multi-dimensional approach captures a more comprehensive biological and clinical profile than single indicators. Consequently, this tool provides clinicians with actionable risk stratification evidence immediately post-surgery, facilitating the optimization of surveillance frequencies and individualized management pathways.

Existing evidence consistently shows that postoperative residual lesions and baseline tumor burden are closely associated with recurrence and survival. However, the predictive stability of single surgical or conventional imaging indicators varies across different medical centers.^15^,^16^ Although the International Federation of Gynecology and Obstetrics (FIGO) staging remains the cornerstone of prognostic evaluation, relying solely on anatomical extent is often insufficient for precision risk stratification. In the present study, the Least Absolute Shrinkage and Selection Operator regression simultaneously retained the FIGO stage, residual lesions, and composite biomarkers, including the Risk of Ovarian Malignancy Algorithm index. This statistical selection inherently demonstrates the independent and complementary predictive value of these variables. Furthermore, the developed nomogram achieved an Area Under the Curve of 0.861 and 0.801 in the training and validation cohorts, respectively. This robust discriminative performance substantially exceeds the historical predictive baseline of single clinical indicators or biomarkers, which typically demonstrate an Area Under the Curve between 0.65 and 0.75. By systematically integrating anatomical staging, surgical quality, and biological activity, this multi-dimensional risk profile provides significant incremental value over traditional single-modality stratification, thereby effectively filling the application gap of existing tools.^15^,^17^

Compared with previous studies, the focus of this research was to advance the prediction window and align it with the actual decision-making nodes of postoperative management. Traditional nomograms primarily target post-recurrence survival, incorporating variables such as recurrence interval, residual lesions, and performance status into the model. Some studies have also utilized perioperative hematological indicators and residual lesions to predict progression-free survival / overall survival in newly treated cohorts.^15^,^17^ This study focuses on recurrence that occurs after surgery, which is consistent with the core clinical concerns following the completion of first-line treatment: determining the frequency of reexamination, decision to introduce enhanced molecular testing, and the management pathway when high-risk signals appear.

Furthermore, the proposed nomogram translates predictive probabilities into actionable clinical pathways, thereby facilitating risk-adapted management. For patients stratified into the high-risk cohort, clinicians may consider the following tailored strategies:

- Intensified surveillance: The conventional 3-month follow-up interval during the first two years post-surgery can be shortened to 1–2 months, incorporating more frequent dynamic evaluations of CA125, HE4, and the ROMA index.
- Enhanced radiological screening: High-risk patients may benefit from expedited imaging schedules, such as biannual contrast-enhanced CT or PET-CT. This approach facilitates the early detection of radiological recurrence, potentially optimizing the therapeutic window for secondary cytoreductive surgery (CRS).
- Tailored maintenance therapy: The high-risk predictors identified by the model (e.g., residual disease > 1 cm, advanced FIGO stage) provide an objective rationale for individualizing first-line maintenance regimens, including the integration of PARP inhibitors or bevacizumab, to effectively delay disease progression.

A similar approach has also been adopted in studies on specific subtypes and molecular pathways. A risk score for clear cell carcinoma, developed and externally validated, supports more refined risk stratification based on biological characteristics.^18^ Additionally, the integration of autophagy-related gene signatures and the immune microenvironment suggest the potential benefit of incorporating molecular pathways into traditional models.^19^

Current evidence supports the use of combined monitoring of biomarkers, rather than relying on any single indicator. The importance of CA125 in diagnosis, efficacy evaluation, and follow-up is unquestionable; however, its sensitivity and specificity in identifying early recurrence have limitations.^20^ The benefits of combining HE4 and CA125 in prognosis and early detection have gradually been established, providing a practical pathway for early identification of high-risk populations after surgery.^21^,^22^

Circulating tumor DNA (ctDNA), regarded as a molecular-level signal of minimal residual disease, often indicates recurrence earlier than imaging and traditional serological markers, and is independently associated with outcomes.^23^ Therefore, combining the model output with dynamic ctDNA monitoring can enable shorter reexamination intervals or trigger intervention assessments for high-risk patients before imaging-detected progression. For low-risk patients with persistently negative ctDNA, this approach is expected to reduce the burden of unnecessary examinations.^20^–^23^

The clinical value of the model is also reflected in its ability to provide early support for treatment pathways in the recurrent phase. Randomized studies on secondary CRS reported conflicting results. No overall survival benefit was observed in the context of high proportions of combined bevacizumab maintenance therapy.^24^ However, progression-free survival was improved when cases eligible for complete resection were strictly selected based on the iMODEL and PET-CT.^25^ This indicates that the benefits of surgery depend on an overall strategy encompassing patient selection, surgical quality control, and integration with systemic therapy. If risk models can identify patients at an elevated risk of relapse shortly before it occurs, combining such models with resectability scores and functional imaging could allow earlier inclusion of potential candidates in multidisciplinary evaluation and treatment pathway planning.^16^,^25^

Regarding future research trajectories, several critical areas warrant further investigation to build upon the current findings. First, while the present model demonstrates robust internal discrimination, prospective validation across independent, multicenter cohorts is imperative to ensure its generalizability across diverse healthcare settings. Second, advancing into the era of precision oncology necessitates the integration of cutting-edge molecular markers, such as BRCA mutations and homologous recombination deficiency status, into the existing predictive framework. Concurrently, incorporating dynamic monitoring technologies, including circulating tumor DNA, holds significant potential for enhancing predictive resolution prior to macroscopic recurrence. Finally, as the therapeutic paradigm shifts, systematically evaluating the impact of novel maintenance therapies, such as poly (ADP-ribose) polymerase inhibitors, on the predictive efficacy of the model will constitute a paramount focus of subsequent studies.

### Limitations:

First, the single-center retrospective design inherently risks selection bias and overfitting. Second, the study period (2020–2022) precluded the systematic integration of evolving maintenance therapies, such as poly (ADP-ribose) polymerase inhibitors. The absence of these adjuvant variables and essential molecular profiles, including BRCA mutation and homologous recombination deficiency status, may compromise model generalizability. Third, defining recurrence as a binary outcome via logistic regression provides intuitive probabilities for fixed-term follow-up but inherently loses valuable temporal data regarding disease progression. Fourth, relying exclusively on a randomly split internal validation cohort without independent external data limits the broad applicability of the nomogram. Furthermore, unstandardized circulating tumor DNA testing protocols and protocol-dependent imaging habitat segmentation collectively affect model reproducibility.^23^,^26^ Future integration into clinical workflows necessitates external prospective validation across multicenter cohorts utilizing time-to-event survival models, such as Cox proportional hazards regression, alongside molecular-stratified recalibration and cost-effectiveness evaluations.^26^,^27^

## CONCLUSION

The risk prediction model for recurrence of EOC based on FIGO staging, differentiation degree, residual lesions, positive ascites cytology, CA125 positivity, and ROMA index positivity, exhibited good predictive performance and applicability. Beyond mere risk screening, this nomogram can serve as an actionable clinical tool to guide personalized follow-up strategies and risk-stratified management pathways for patients after primary CRS.

### Authors’ contributions:

**WZ:** Literature search, study design and manuscript writing.

**YS, ZW and SY:** Data collection, data analysis and interpretation. Critical Review

**WZ:** Manuscript revision and validation and is responsible for the integrity of the study.

All authors have read and approved the final manuscript.
